# Supplementary material for: An apparent lack of synergy between degradative enzymes against Staphylococcus aureus biofilms
Source: bioRxiv. 2023 Oct 5:2023.10.05.561034. Preprint. [Version 1] doi: 10.1101/2023.10.05.561034 (PMC10592981; doi:10.1101/2023.10.05.561034)
Supplement: Supplement 1 [file NIHPP2023.10.05.561034v1-supplement-1.pdf]

bioRxiv preprint doi: https://doi.org/10.1101/2023.10.05.561034; this version posted October 5, 2023. The copyright holder for this preprint (which was not certified by peer review) is the author/funder, who has granted bioRxiv a license to display the preprint in perpetuity. It is made available under aCC-BY 4.0 International license.

Supplement

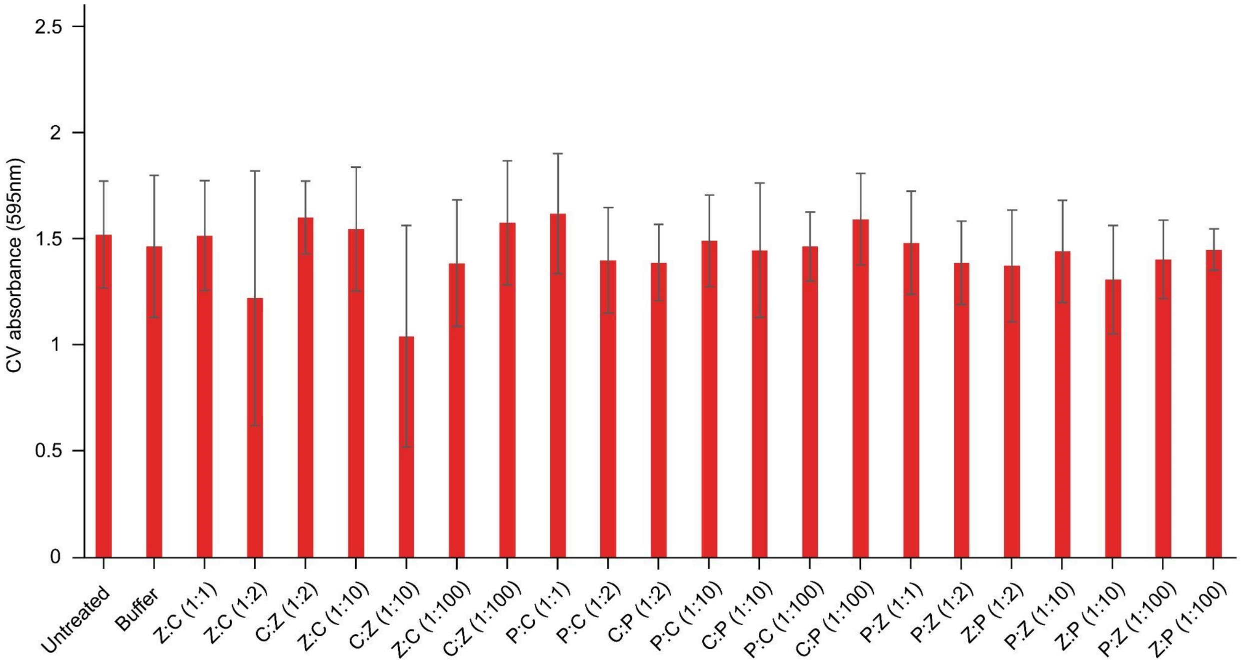

Figure S1. No synergy between different ratios of enzymes at a low concentration *S. aureus* biofilms grown on polystyrene were treated with mixed enzyme combinations for one hour. Enzyme combinations were mixed at ratios of 1:1, 1:2, 1:10, and 1:100 with total enzyme concentration at 0.06 mg mL^{-1}. Wells were stained with 0.1% crystal violet, and the absorbance was measured at 595 nm (P = pepsin, Z = zymolyase 20T, C = cellulase) (n = 3).

bioRxiv preprint doi: https://doi.org/10.1101/2023.10.05.561034; this version posted October 5, 2023. The copyright holder for this preprint (which was not certified by peer review) is the author/funder, who has granted bioRxiv a license to display the preprint in perpetuity. It is made available under aCC-BY 4.0 International license.

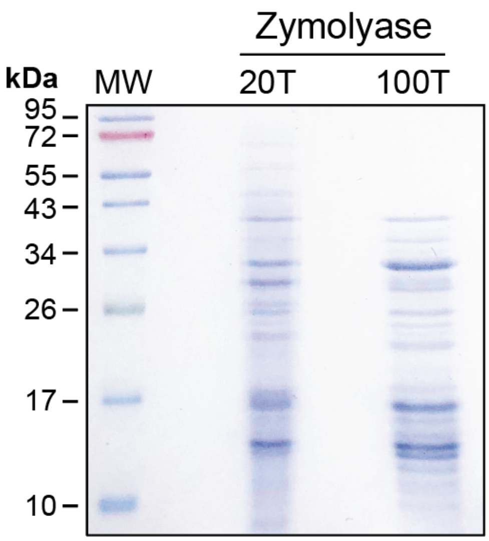

Figure S2. Analysis of Zymolyase 20T and 100T by SDS PAGE. 10 ug of zymolyase 20T and 100T were analyzed by 10% SDS PAGE. The molecular weight marker used in lane 1 was the Color Prestained Protein Standard, Broad Range (BioRad).
